# Horse–, training– and race–level risk factors for palmar/plantar osteochondral disease in the racing Thoroughbred

**DOI:** 10.1111/evj.12038

**Published:** 2013-02-20

**Authors:** G L Pinchbeck, P D Clegg, A Boyde, E D Barr, C M Riggs

**Affiliations:** * Institute of Infection and Global HealthFaculty of Health and Life Sciences, University of LiverpoolUK; † Department of Musculoskeletal BiologyFaculty of Health and Life Sciences, University of LiverpoolUK; ‡ Biophysics, Oral Growth and Development, Barts and The London School of Medicine and DentistryUK; § Bell Equine Veterinary ClinicMereworth, Maidstone, Kent, UK; # Department of Veterinary Clinical Services, Hong Kong Jockey ClubChina

**Keywords:** horse, metacarpo/metatarsophalangeal joint, osteoarthritis, palmar/plantar condyle, racing intensity, risk factors

## Abstract

**Reasons for performing study** Palmar/plantar osteochondral disease (POD) is a common, debilitating condition in Thoroughbred racehorses; however, training- and racing-related factors associated with this disease are unknown.

**Objectives** To determine horse-, racing- and training-related risk factors for POD. The general hypotheses were that early training and racing, and increased intensity of racing and training, lead to increased severity of POD.

**Methods** The metacarpo/metatarsophalangeal joints of 164 Thoroughbred racehorses were examined at *post mortem* and graded for third metacarpal and metatarsal POD. The relationships between training- and racing-related factors and grade of POD in each condyle were determined using multilevel, multivariable, ordinal logistic regression models.

**Results** A total of 1288 condyles were graded. Factors associated with higher grades of POD were the total lifetime number of races, an increase in gallop sessions in the previous season, racing before import to Hong Kong and an increase in the number of short (8–16 weeks) between-race intervals per season. Horses in their first racing season were more likely to have lower POD grades, while horses that had a long between-race interval (greater than 16 weeks) in the season prior to euthanasia were also more likely to have lower POD grades. Lower POD grades were significantly more likely as days since last race increased up to 400 days. Age at first race was not significantly associated with grade of POD.

**Conclusions and potential relevance** Cumulative racing exposure and training intensity in the previous season were associated with higher grades of POD, supporting the hypothesis that the disease is due to repetitive loading. Longer between-race intervals and increased time since racing were associated with lower POD grades, which may indicate that lesions heal. Further work is required to enable optimisation of racing and training programmes to reduce the frequency and severity of this disease.

## Introduction

Palmar/plantar osteochondral disease (POD) is a degenerative condition affecting the distal condyles of the third metacarpal and metatarsal bones (Mc/MtIII) and is common in the Thoroughbred racehorse 1. Grossly, the lesions appear as small (2–4 mm diameter), ovoid defects in the palmar or plantar articular surface of the condyle. Early findings include a focus of bluish discolouration of the subchondral bone with grossly normal articular cartilage, while more severe changes include physical disruption of the subchondral bone associated with varying degrees of pathology of the overlying articular cartilage, including ulceration and collapse 1. The condition is believed to be due to injury of subchondral bone associated with repetitive high strains and strain rates in bone of the distal condyles of Mc/MtIII during high-speed racing and training 2. The clinical condition of pain associated with subchondral bone injury of the distal condyles of Mc/MtIII has become well recognised in most racing breeds, including Thoroughbreds 3–4; however, training- and racing-related risk factors associated with the disease and with its severity have not been evaluated previously and yet are essential in order to develop strategies to prevent the onset and progression of POD.

This study aimed to determine horse-, racing- and training-related risk factors for POD in a population of flat-racing Thoroughbreds in Hong Kong. A number of hypotheses were formed prior to the study based on previous literature 2,3 and the authors' knowledge of the clinical condition and its presentation in racing Thoroughbreds. These were as follows: 1) POD is more severe in horses that commence high-impact training or racing as 2-year-olds; 2) severity of POD is increased in horses raced over longer distances; 3) severity of POD is increased in horses raced and trained more frequently; 4) severity of POD is increased in heavier horses due to increased stress on joint tissues; 5) severity of POD is greater in those horses that have more short rest periods in between racing; 6) swimming as part of the training regimen has a protective effect on severity of POD (due to less impact damage); 7) POD is more prevalent in certain bloodlines; and 8) winnings from racing are reduced in horses with increased grades of POD.

## Methods

Material was examined from 166 male Thoroughbred racehorses that were either in, or had been retired from, active race training at the Hong Kong Jockey Club from 7 March 2005 to 25 March 2009. The methods of material collection, reasons for death/euthanasia, scoring of POD at *post mortem* and associated pathological findings for the first 64 horses are described in detail by Barr *et al*. 1 and for the further 102 by Pinchbeck *et al*. 5 In brief, the distal articular surface of Mc/MtIII was examined by gross observation at *post mortem* predominantly by one observer (C.M.R.). A scoring system (Table 1) was developed, and each condyle was assigned a score for POD.

**Table 1:** Scoring system for grading of palmar/plantar osteochondral lesions on gross observation at *post mortem*, and numbers and percentages of each grade in 1288 condyles from 164 flat-racing Thoroughbreds in Hong Kong

Grade of POD	Description	Number of condyles of each grade (n = 1288)	Percentage of condyles of each grade (n = 1288)	95% confidence interval
0	No evidence of POD	727	56.4	53.7–59.2
1	Discolouration (bruising) of subchondral bone only, with no disruption or minimal disruption of overlying articular cartilage	364	28.3	25.8–30.8
2	Discolouration (bruising), with mild to moderate disruption of articular cartilage	151	11.7	10.1–13.6
3	Established POD lesions. Discolouration and disruption/collapse of articular surface	45	3.5	2.6–4.6

Abbreviation: POD = palmar/plantar osteochondral disease.

Of these 166 horses, one never entered training or racing in Hong Kong (and therefore was not included in analysis) and one had an identification number that could not be traced in the health, training and racing performance database and was also excluded.

Detailed data for the remaining 164 horses were extracted from computerised records held by the Hong Kong Jockey Club and imported into an Access database. The computerised records held by the Hong Kong Jockey Club are part of an official Racing Information System established in 1992. The Racing Information System was extended to include veterinary records in 1996, which was subsequently replaced by a dedicated Veterinary Management Information System in March 2006. All aspects of racing and training are strictly regulated at the Hong Kong Jockey Club, and clinicians have a responsibility to record all significant clinical findings and all medications administered or prescribed promptly into the Veterinary Management Information System.

Racing and training variables examined are shown in Tables S1 and S2, respectively. Five horses entered training but never raced, either in Hong Kong or prior to import, and were therefore excluded from analyses of racing exposure variables. In addition, 6 horses started their training career prior to August 1996 when the training data collection system commenced, hence incomplete training data were available and they were excluded from the training data analyses.

In most horses, all 8 condyles were examined (medial/lateral, left/right and front/hind); however it was not possible to score accurately all features of the condyles in every joint on account of extensive pathology associated with acute trauma, and data for 24 individual condyles from 12 different horses were therefore missing.

### Data analysis

Sample size estimates allowing for within-horse clustering were performed using software MLPowSim 6 (for sample size calculations for random effect models via simulation). This was based on a binary outcome (ignoring the ordinal outcome) due to software limitations. Sample size estimates were performed after the initial study on 64 horses 1. This allowed an estimate of within-horse variance of 3.67 (s.e. 0.68). Sample size estimates showed that 150 horses with 8 condyles per horse would give over 90% power with 95% confidence to detect an odds ratio of 2.5 or greater for exposures with 20% prevalence.

To identify highly correlated variables (correlation coefficient >0.8), Pearson's correlation coefficient for normally distributed continuous data and Spearman's rhos for other data were used. Where variables were highly correlated, the most biologically meaningful, easily interpretable or the most significant variables were used in the multivariable model.

To estimate the proportion of variance attributable to trainer, sire, dam and sire of dam, 3-level intercept-only, ordered proportional odds models were used to estimate the variance level, and approximations of the intraclass correlation coefficients were calculated using a latent-variable approach 7, which assumes that the level one variance on the logit scale is (π^2^)/3.

We chose to examine an ordinal outcome of POD in this study to reflect differing severities of POD and increase the power of the study. The association of variables with the ordinal outcome of POD grade (*0–3*), with POD *grade 0* as the reference category, was performed using a multilevel, ordered proportional odds model with a logit link function 8, with horse included as a random effect to allow for the clustering of outcomes at the individual condyle level within horse. Continuous variables were assessed for linearity either by categorisation and assessing the relationship or by fitting polynomials and assessing the significance of these 9.

Variables with P<0.3 on univariable analysis were considered for inclusion in a multivariable model. The final multivariable model was initially built as a 2-level model incorporating clustering at the horse level. Due to the small numbers in POD *grades 2* and *3*, these were combined, leading to an ordinal outcome of POD grade of *0*, *1* or *2/3*. Given that both *grade 2* and *3* scores of POD have obvious chondral pathology and marked subchondral bone resorption 1, it was felt reasonable to combine these groups, because both demonstrated advanced forms of this disease. The model was built using a stepwise forward and backward elimination process 10. Variables with a termwise Wald test P<0.05, or that showed evidence of confounding assessed by change in the coefficient of other variables of greater than 30%, were retained in the model. Random coefficients models (random slopes) were assessed for all variables in the final model to determine whether the effects varied by horse. Two-way interaction terms were tested between all plausible biological terms in the final model. The assumption of proportional odds was assessed for all variables in the final model by formally testing (using the Wald test) whether coefficients fitted individually for each response category were significantly different from one another 8.

Initial univariable and multivariable calculations were performed using penalised quasi-likelihood estimates with 2nd order Taylor series expansion 8. However, estimates for the final model were obtained using a Monte Carlo Markov Chain with Metropolis Hasting sampling with diffuse priors, a burn-in period of 10,000 iterations and a run of 500,000 iterations. The number of iterations required and evaluation of model convergence was determined by examination of the Monte Carlo Markov Chain diagnostics 11.

Data were analysed using SPSS version 16.03a and multilevel models used the MLwiN statistical software package (MLwiN Version 2.184b).

## Results

A total of 1288 condyles were examined, and the severity of POD was graded (Table 1). Correlation analysis showed that a number of racing variables were highly correlated (correlation coefficient >0.8). These included a number of variables relating to the duration, distance and intensity of racing and training. The total races in the horse's lifetime, total races in Hong Kong, the total number of racing seasons, the total number of races on turf and the lifetime total race distance were all highly correlated with each other. In addition, the number of between-race intervals of 2, 2–4 and 4–8 weeks was also highly correlated with these variables relating to racing intensity. The peak weight and average weight of the horse were also highly correlated.

A similar pattern was seen in the training data, where the total career days, the total number and total distance of gallops over the lifetime, the total number and total distance of trots over the lifetime and the number of seasons in training were all highly correlated with each other. The number of gallops in the last season was correlated with the number of last-season between-gallop intervals of <1 week. The number of days since retirement and the number of days since last race to date of euthanasia were also highly correlated.

Intercept-only models showed that there was significant clustering of POD pathology within horse (variance estimate [v.e.] 3.8, s.e. 0.53), indicating approximately 54% clustering. The 164 horses were trained (trainer in season before euthanasia or retirement) by a total of 31 trainers. After allowing for within-horse clustering, there was little clustering within trainer (v.e. 0.5, s.e. 0.36), indicating approximately 6% clustering. There were 127 different sires, 163 different dams and 128 different grandsires (the grandsire of one horse was unknown). The clustering of POD within dam and sire of dam were both negligible (coefficients <0.0001); however, there was a small amount of clustering within sire (v.e. 1.02, s.e. 0.98), indicating approximately 13% clustering. Initial univariable and multivariable analyses were therefore conducted, allowing for clustering within horse only, but the final model also included sire effects.

### Univariable analysis of horse- and race-level variables

Univariable analysis identified a number of racing variables that had significant associations with grade of POD (Table S1). Those significantly (P<0.05) associated with an increased probability of higher POD grades were as follows: racing before import into Hong Kong; greater age at retirement; increasing number of races in Hong Kong, in lifetime, on turf, at either Happy Valley or Sha Tin racecourse; increasing number of racing seasons; increasing number of races per season; increasing total lifetime race distance and increasing average race distance over career or in the most recent season; increasing race earnings over lifetime; and increasing average number of between-race intervals of up to 16 weeks.

Those variables associated with a decreased probability of higher POD grades were fewer and included the following: starting racing career at age of 4 years or greater; increasing number of days since retirement or last race and the time of death or euthanasia; and an increasing number of between-race intervals of greater than 16 weeks duration during the current season.

Variables with no significant effect included import age or age of first race in Hong Kong, earnings and performance in the most recent racing season and the horse's average or peak weight during its career.

### Univariable analysis of training variables

Univariable analysis (Table S2) showed that a number of training variables also had significant associations with POD grade. Those associated with an increased probability of having higher POD grades were the total number of career days, the total number and distance of gallops and barrier trials over the training career of the horse, the average gallops per season and the total number of gallops in the season prior to euthanasia or retirement, swimming in training and an increase in the average number of between-gallop intervals per season over the training career of <1, 1–2 and 2–4 weeks. The total number of between-gallop intervals of <1 and 1–2 weeks out of training in the last season before retirement or euthanasia was also significant. There were no significant variables associated with a decreased probability of higher POD grade categories. Variables with no significant effect included age at first track work and number of gallop intervals of >4 weeks, averaged either over lifetime or in last season.

### Multivariable analysis

The final multivariable model (Table 2) identified a number of variables that were significantly associated with POD grade. The number of days from last race to the date of euthanasia was not linearly associated with the outcome, and the best fit was provided by a piecewise term allowing the risk to decrease up to 400 days, with further time out of work having no further effect. This variable violated the proportional odds assumption and therefore separate coefficients were fitted for each POD category. This showed that there was a greater effect of an increase in days since last race across POD categories POD *grade 0* to POD *grade 1* and a lesser effect across POD *grade 2/3* to POD *grade 1*. In addition, as the total number of races in Hong Kong in the horse's career increased, the risk of having a higher grade of POD increased. Likewise, an increasing total number of gallops in the season prior to euthanasia (or retirement) was associated with higher probability of higher grades of POD. Horses that had raced before import showed an increased probability of having higher POD grades, while those in the first racing season were more likely to have lower POD grades compared with horses in later seasons. Horses that had a between-race interval of >16 weeks in the season prior to euthanasia or retirement had greater probability of having lower POD grades. However, an increasing average number of between-race intervals of 8–16 weeks per season over the training career increased the risk of higher POD grades. The variance estimate for horse decreased, indicating that this model explains some of the within-horse clustering, but there appeared to be some remaining clustering, and there was also a small amount of within-sire clustering.

**Table 2:** Multilevel, multivariable ordered proportional odds models of factors associated with grade of palmar/plantar osteochondral disease in 153 Hong Kong racehorses

Variable	Coefficient	Standard error	Odds ratio	Lower 95% CI	Upper 95 CI	P value
POD = *grade 1* intercept	-3.81	0.81	-	-	-	-
POD = *grade 2/3* intercept	-6.55	0.83	-	-	-	-
Sire-level variance	0.79	0.44	-	-	-	-
Horse-level variance	1.37	0.48	-	-	-	-
Fixed-effect variables						
POD *grade 1*: days from last race to date of death/euthanasia (up to 400 days)*	-0.007	0.001	0.99	0.99	0.99	<0.001
POD *grade 2/3*: days from last race to date of death/euthanasia (up to 400 days)*	-0.002	0.001	1.00	1.00	1.00	0.3
Average number of between-race intervals of 8–16 weeks, per season, over training career	0.89	0.36	2.43	1.19	4.96	0.01
Total number races in Hong Kong over lifetime (range 1–74)	0.04	0.01	1.04	1.02	1.06	<0.001
Total number gallops in most recent season before euthanasia or retirement	0.02	0.009	1.02	1.01	1.04	0.01
Season number						
First						
Second or later	1.96	0.74	7.13	1.69	30.10	0.007
Raced before import						
No						
Yes	0.66	0.30	1.93	1.08	3.46	0.02
Between-race interval of >16 weeks in the most recent season prior to euthanasia or retirement						
No						
Yes	-1.47	0.64	0.23	0.07	0.80	0.03

Abbreviations: CI = confidence interval; and POD = palmar/plantar osteochondral disease. Outcome is POD *grade 0–2/3* (*grade 3* was combined with *grade 2*). Palmar/plantar osteochondral disease *grade 0* was treated as the reference category; therefore, positive coefficients (odds ratios greater than one) indicate that the probability of being in the higher categories is increased and negative coefficients (odds ratios less than one) indicate that the probability of being in the higher categories is decreased.

* The variable ‘days since last race’ was fitted as a piecewise term, allowing a linear decrease up to 400 days and then no change. This variable violated the proportional odds assumption and therefore separate coefficients for each category were included. P values are from the Wald chi-squared test.

The posterior distributions of the variables included in the model are not shown, but the fits were smooth and regular and all chains mixed well for all fixed-effect variables, thus demonstrating good convergence. Prohibitively long chain lengths seemed to be required to give certainty about the 97.5th and 2.5th quantiles of the posterior distributions for the random effect of sire. However, according to the Brooks–Draper statistic, sufficient iterations were performed to give certainty about estimates for the means of both horse and sire.

## Discussion

Degenerative lesions of the subchondral bone forming the distal condyles of Mc/MtIII (called here ‘POD’) have been recognised as a common cause of lameness in racehorses for over 30 years. This is the first study to investigate factors that influence prevalence of this condition. The principal finding is that POD is associated with racing and training exposures; increased number of races in a lifetime, increased training gallops in the most recent season and horses that had raced for more than one season all increased the risk of higher grade POD. This observation supports the hypothesis that the disease is due to injury of subchondral bone after it is subjected to prolonged, cyclical loading at high stress levels that arise when the horse gallops. In addition, high strain rates, associated with high-speed locomotion, will be more likely to inflict damage on the bone matrix and injure bone cells. Total distances galloped and raced were also highly significant on univariable analysis but were highly correlated with number of races and therefore did not remain in the final multivariable model.

Horses that had raced before import to Hong Kong were also at increased risk of higher grades of POD. This may reflect the fact that these horses performed more intensely at a high level at a younger age to achieve the performance criteria required for import, or that they had worked on different training/racing surfaces. It is also conceivable that overseas vendors may, in some cases, sell horses because they become suspicious of a subclinical condition and wish to capitalise on the horse's value before the condition becomes clinically apparent. However, although we hypothesised that POD would be more severe in horses raced earlier, before the skeleton matures, the age at first race and age at first track work were not significant in the final model, and it appears that cumulative exercise may be more important than age at first exercise. A recent study has shown that early racing may have a beneficial effect on career longevity, with horses that raced as 2-year-olds having more race starts and more years racing than those first raced as 3-year-olds or older. This may be due to improved musculoskeletal health 12.

We hypothesised that earnings and performance would be lower in horses with higher grades of POD, due to pain, but this was not supported by the data. There was no association with earnings and performance in the most recent season, and on univariable analysis the earnings and performance over the lifetime were higher in horses with higher POD grades; however, this association is most likely to be related to increased racing intensity in horses with POD and it did not remain in the final model. Conversely, in a prospective study, Trope *et al*. 13 did demonstrate fewer starts, less prize money and less prize money per start in horses with moderate to marked increased palmar/plantar radioisotope uptake compared with control animals.

Swimming as part of the training regimen did not remain in the final model, although on univariable analysis swimming increased the risk of POD. This may be because trainers are more likely to swim horses with clinical lameness, and swimming is therefore likely to be an effect of POD rather than a cause. Given that the condition is associated with recurrent overload of the joint, it was surprising that neither average nor peak weight of the horse was significant on univariable analysis; however, there was little variation in weights between Thoroughbred racehorses in this study and this may have prevented detection of an effect.

Horses that had had an interval off work of >16 weeks in the season prior to euthanasia or retirement and horses with a greater number of days since the last race to the date of euthanasia were more likely to have lower POD grades. This may reflect the possibility that lesions can heal following appropriate rest from training and racing. However, this effect was more marked in lower-grade lesions, suggesting that *grade 1* lesions may be better able to heal, which is logical because there is minimal structural damage and no disruption of articular surface in such cases. This emphasises the importance of prompt diagnosis of this condition, although definitive diagnosis of low-grade POD lesions is difficult to achieve without the use of specialised imaging modalities, such as magnetic resonance imaging or computed tomography. Further longitudinal studies are now required to confirm whether low-grade POD lesions do progress (i.e. they reflect early stages of a syndrome), and to determine up to what stage lesions can be reversed. In addition, there is a need for simpler methods to detect early lesions and for studies to determine the optimal rest periods to allow lesions to heal while causing minimal disruption to a horse's racing career.

Contrary to the effect of prolonged rest periods, an increased number of short-duration rest periods (average number of intervals of 8–16 weeks between races over the career of the horse) was associated with increased risk of higher POD grades. It is not possible to determine from this study whether this reflects cause or effect; horses may have multiple short intervals off work because they suffer recurrent lameness due to POD. If the interval is too short, healing of the POD lesion may be incomplete and the lesion fails to resolve. In fact, returning to training while lesions are still undergoing the resorptive phase of bone healing may lead to more severe changes, such as saucer fracture of subchondral bone or collapse of articular surface into cavities created by focal, intense remodelling 14.

The large amount of clustering within horse was expected owing to the sampling of all 8 condyles from the majority of horses. Many of the exposures assessed in this study are at the level of the horse (i.e. are equivalent on all limbs), and inclusion of horse as a random effect is therefore necessary to avoid incorrect estimation of the significance of individual variables. Estimating the proportion of variance due to levels of clustering can also allow targeting of areas in current research 15–16. There were no dam effects, and this is not surprising owing to the almost equal number of dams to horses. However, despite the large number of sires, there was still an independent sire effect, suggesting that there is a small proportion of variation in POD grades that is independently attributable to sire. This may be a reflection of a different genetic basis of exercise-induced injury. Previous studies have shown some heritability for other orthopaedic disease of equines, such as osteochondrosis and palmar/plantar osseous fragments 17. Whether genetic effects are due to matrix alterations in osteochondral tissues or are a consequence of specific conformational traits that may have some heritable basis would be an interesting area of study.

Trainer effects were relatively small in this study, and the addition of the fixed effects further reduced the clustering within trainer. Others have found that there were significant trainer effects for tendon strain injury 15–18 and dorsometacarpal disease 19, as well as for performance 20. Trainers in Hong Kong all share the same training facilities, are based in the same complex and have access to the same tracks. This generates a degree of homogeneity that is absent from most other training environments in the world.

The Racing Information System database in Hong Kong contains a large number of variables available for analyses, but many of these were correlated, and the decision of which to include in multivariable models was based mainly on which was the most biologically meaningful or easiest to interpret. The Hong Kong Jockey Club provides a unique environment in which to conduct a study such as this. Once imported to Hong Kong, horses nearly always complete their racing careers at the Hong Kong Jockey Club; all horses that die or are destroyed are subjected to compulsory *post mortem* examination; the Department of Veterinary Clinical Services is the only provider of veterinary services, and the level of confidence in clinical data is extremely high owing to regulatory requirements and the strict reporting structure within the Hong Kong Jockey Club.

This study was cross-sectional in nature, and euthanasia occurred for a number of reasons 1–5, many of which were related to the musculoskeletal system, which may have introduced some bias into these results. It is difficult to know which direction this bias may take, because horses without POD but with other musculoskeletal injuries may have had a modified or reduced training regimen. Conversely, they may have had more intense racing and training exposure that predisposed them to musculoskeletal injury. The fact that it is still possible to grade this condition only at *post mortem* is a severe handicap to studies that are required to answer these questions. The development of imaging techniques that can identify and grade POD in the live horse will improve the ability to study this disease and the selection of appropriate control populations 21.

In conclusion, this study has shown that the amount of cumulative racing exposure and training intensity in the previous season were associated with higher grades of POD, thereby supporting the hypothesis that the disease is due to repetitive high-stress loading of subchondral bone. In addition, longer between-race intervals and time since last race were associated with lower POD grades, which provide evidence that lesions may heal. Further work is required to identify thresholds in the intensity or duration of training and racing that lead to formation of POD lesions. This will enable trainers to optimise a horse's career while reducing the risk and/or severity of this common and debilitating disease.

## Authors' declaration of interests

No competing interests have been declared.

## Sources of funding

This project was funded by the Horserace Betting Levy Board and the Hong Kong Jockey Club.

## Manufacturers' addresses

^a^SPSS Inc., Chicago, Illinois, USA.

^b^Centre for Multilevel Modelling, University of Bristol, Bristol, UK.

## Supporting Information

Table S1 Horse and race variables examined and their relationship with grade of palmar/plantar osteochondral disease (POD) from univariable multilevel, multinomial, ordered proportional odds models in 164 Hong Kong racehorses.

Table S2 Training variables examined and their relationship with grade of palmar/plantar osteochondral disease (POD) from univariable multilevel, multinomial, ordered proportional odds models in 158 Thoroughbred racehorses in Hong Kong.
