# Supplementary material for: Horse–, training– and race–level risk factors for palmar/plantar osteochondral disease in the racing Thoroughbred
Source: Equine Vet J. 2013 Feb 20;45(5):582–6. doi: 10.1111/evj.12038 (PMC3883097; doi:10.1111/evj.12038)
Supplement: Table S1 — Horse and race variables examined and their relationship with grade of palmar/plantar osteochondral disease (POD) from univariable multilevel, multinomial, ordered proportional odds models in 164 Hong Kong racehorses. [file evj0045-0582-sd1.doc]

**Supplementary Item 1:**

Horse and race variables examined and their relationship with grade of POD from univariable multilevel, multinomial, ordered proportional odds models in 164 Thoroughbred racehorses Hong Kong. The outcome is categories of POD grade 0 to grade 3. POD grade 0 was treated as the reference category therefore negative coefficients (odds ratio <1) indicate that the probability of being in the lower categories is increased and positive coefficients (odds ratios >1) indicate that the probability of being in the higher categories is increased. For brevity the separate intercepts for each grade of POD and the within horse variance estimate for each analysis are not shown, however horse was included as a random effect in all analyses. Five horses entered training but never raced either in HK or prior to import therefore these 5 are excluded from analyses of racing exposure variables.

| **Variable** | **Coefficient** | **Standard Error** | **Odds ratio** | **Lower 95% CI** | **Upper 95 CI** | **P -value** |
| --- | --- | --- | --- | --- | --- | --- |
| **All 164 horses** |  |  |  |  |  |  |
| Import age |  |  |  |  |  |  |
| 2 years | Ref |  | Ref |  |  |  |
| 3 years | 0.49 | 0.36 | 1.64 | 0.80 | 3.33 | 0.2 |
| 4 years | 0.96 | 0.57 | 2.62 | 0.85 | 8.01 | 0.1 |
| Number racing seasons in Hong Kong |  |  |  |  |  |  |
| 0 | Ref |  | Ref |  |  |  |
| 1 | -1.33 | 1.38 | 0.26 | 0.02 | 3.92 | 0.3 |
| 2 | 1.13 | 1.11 | 3.09 | 0.35 | 27.45 | 0.3 |
| 3 | 1.62 | 1.11 | 5.05 | 0.57 | 44.46 | 0.1 |
| 4 | 2.56 | 1.12 | 12.90 | 1.44 | 115.84 | 0.02 |
| 5 | 2.65 | 1.13 | 14.21 | 1.55 | 130.16 | 0.02 |
| 6 | 1.69 | 1.20 | 5.39 | 0.52 | 56.32 | 0.2 |
| 7 | 1.18 | 1.41 | 3.25 | 0.21 | 51.60 | 0.4 |
| 8 | 4.49 | 1.70 | 89.12 | 3.19 | 2490.01 | 0.008 |
|  |  |  |  |  |  |  |
| Raced before import into Hong Kong |  |  |  |  |  |  |
| No |  |  |  | Ref |  |  |
| Yes | 1.06 | 0.35 | 2.89 | 1.45 | 5.74 | 0.002 |
| Country of import |  |  |  |  |  | 0.7 |
| Australia | Ref |  | Ref |  |  |  |
| New Zealand | 0.27 | 0.40 | 1.31 | 0.59 | 2.88 | 0.3 |
| USA | 0.40 | 1.02 | 1.49 | 0.20 | 11.05 | 0.09 |
| GB | -0.38 | 0.54 | 0.68 | 0.24 | 1.97 | 0.5 |
| Ireland | 0.86 | 0.77 | 2.36 | 0.53 | 10.62 | 0.09 |
| South Africa | 0.36 | 1.18 | 1.44 | 0.14 | 14.64 | 0.07 |
| Other (Brazil, Arg, France, Canada) | -0.68 | 1.26 | 0.51 | 0.04 | 6.01 | 0.2 |
| Retired age (cont 2-10 years) | 0.40 | 0.10 | 1.49 | 1.22 | 1.82 | <0.001 |
| Total no. race HK (cont 0-74) | 0.05 | 0.01 | 1.05 | 1.03 | 1.07 | <0.001 |
| Total no. races lifetime (cont 0-74) | 0.05 | 0.01 | 1.05 | 1.03 | 1.07 | <0.001 |
| Total races at Happy Valley course (cont 0-35) | 0.08 | 0.02 | 1.08 | 1.04 | 1.13 | <0.001 |
| Total races Sha Tin course (cont 0-65) | 0.05 | 0.01 | 1.05 | 1.03 | 1.08 | <0.001 |
| No of races before import to HK cont (0-13) | 0.13 | 0.05 | 1.14 | 1.03 | 1.26 | 0.01 |
| Total no. races in HK on all weather (cont 0-34) | 0.10 | 0.03 | 1.10 | 1.04 | 1.16 | 0.001 |
| Total no. races in HK on Turf (cont 0-73) | 0.05 | 0.01 | 1.05 | 1.03 | 1.07 | <0.001 |
| Days since retirement (cont 0-6528) to date of euthanisia or death | -0.13 | 0.04 | 0.88 | 0.81 | 0.96 | 0.002 |
| Days =0 | Ref |  | Ref |  |  |  |
| Days = 1-100 | -0.10 | 0.43 | 0.91 | 0.39 | 2.09 | 0.8 |
| Day >100 | -2.06 | 0.59 | 0.13 | 0.04 | 0.40 | <0.001 |
|  |  |  |  |  |  |  |
| **N = 159 (Excludes 5 horses that never raced)** |  |  |  |  |  |  |
| Age first raced in Hong Kong |  |  |  |  |  |  |
| 2 years |  |  | Ref |  |  |  |
| 3 years | 0.39 | 0.54 | 1.47 | 0.51 | 4.26 | 0.5 |
| 4 years | 0.75 | 0.59 | 2.11 | 0.66 | 6.77 | 0.2 |
| 5 or 6 years | -1.88 | 1.47 | 0.15 | 0.01 | 2.70 | 0.2 |
| Age first raced life |  |  |  |  |  |  |
| 2 years |  |  | Ref |  |  |  |
| 3 years | -0.33 | 0.36 | 0.72 | 0.36 | 1.46 | 0.4 |
| 4 or greater | -1.52 | 0.62 | 0.22 | 0.06 | 0.74 | 0.01 |
|  |  |  |  |  |  |  |
| Age first raced HK (in years; cont 2-6) | 0.10 | 0.25 | 1.10 | 0.67 | 1.81 | 0.7 |
| Age first race life (in years; cont 2-6) | -0.60 | 0.26 | 0.55 | 0.33 | 0.91 | 0.02 |
| Total number racing seasons in HK (cont 1-8) | 0.40 | 0.11 | 1.49 | 1.21 | 1.84 | <0.001 |
| No. races most recent season (cont 1-18) | 0.07 | 0.05 | 1.07 | 0.97 | 1.18 | 0.2 |
| Average number races per season (cont 1-12.3) | 0.32 | 0.06 | 1.38 | 1.22 | 1.56 | <0.001 |
| Total lifetime race distance (in 1000 metres) (cont 1- 110) | 0.03 | 0.01 | 1.03 | 1.02 | 1.05 | <0.001 |
| Average race distance (in 100 metres) (cont 10-20.3) | 0.30 | 0.08 | 1.36 | 1.16 | 1.59 | <0.001 |
| Average race distance most recent season (in 100 metres) (cont 10-20) | 0.25 | 0.07 | 1.29 | 1.12 | 1.48 | <0.001 |
| Days from last race to date of euthanasia or death (cont 0 – 6530) | -0.14 | 0.04 | 0.87 | 0.80 | 0.94 | <0.001 |
| Days = 0 | Ref |  | Ref |  |  |  |
| Days= 1-100 | -0.19 | 0.63 | 0.83 | 0.24 | 2.82 | 0.8 |
| Days = >100-365 | -1.43 | 0.68 | 0.24 | 0.06 | 0.91 | 0.04 |
| Days= >365 | -2.58 | 0.83 | 0.08 | 0.01 | 0.38 | <0.001 |
| Average earnings in most recent season (in 100,000 HK$) | -0.002 | 0.21 | 1.00 | 0.66 | 1.51 | 0.9 |
| Total earnings most recent season (in 100,000 HK $) | 0.03 | 0.05 | 1.03 | 0.93 | 1.14 | 0.6 |
| Total stakes won (in 100,000 HK$) | 0.02 | 0.01 | 1.02 | 1.00 | 1.04 | 0.02 |
| Total lifetime earnings (in 1000 HK$) | 0.02 | 0.01 | 1.02 | 1.01 | 1.04 | 0.01 |
| Average earnings per season raced over life (in 100 HK$) | 0.13 | 0.05 | 2.72 | 1.14 | 1.05 | 0.009 |
| Between-race intervals current season <2 weeks | -0.03 | 0.14 | 0.97 | 0.74 | 1.26 | 0.8 |
| Between-race intervals current season 2-4 weeks | 0.00 | 0.10 | 1.00 | 0.83 | 1.21 | 0.9 |
| Between-race intervals current season 4-8 weeks | 0.41 | 0.12 | 1.51 | 1.18 | 1.92 | 0.001 |
| Between-race intervals current season 8-16 weeks | 0.57 | 0.31 | 1.76 | 0.96 | 3.21 | 0.07 |
| Between-race intervals current season >16 weeks | -1.92 | 0.80 | 0.15 | 0.03 | 0.70 | 0.02 |
| Between-race intervals <2 weeks average per season over lifetime | 0.56 | 0.19 | 1.75 | 1.22 | 2.52 | 0.002 |
| Between-race intervals 2-4 weeks average per season over lifetime | 0.42 | 0.12 | 1.52 | 1.21 | 1.90 | <0.001 |
| Between-race intervals 4-8 weeks average per season over lifetime | 0.78 | 0.17 | 2.18 | 1.58 | 3.01 | <0.001 |
| Between-race intervals 8-16 weeks average per season over lifetime | 1.58 | 0.38 | 4.85 | 2.28 | 10.29 | <0.001 |
| Between-race intervals life >16 weeks average per season over lifetime | -1.03 | 0.60 | 0.36 | 0.11 | 1.17 | 0.09 |
| No. first place last season (cont 0-3) | -0.32 | 0.25 | 0.72 | 0.44 | 1.18 | 0.8 |
| No. 2^nd^ place last season (cont 0-4) | 0.31 | 0.26 | 1.37 | 0.83 | 2.26 | 0.2 |
| No. 3^rd^ place last season (cont 0-3) | 0.14 | 0.25 | 1.15 | 0.70 | 1.88 | 0.6 |
| No. 4^th^ place last season (cont 0-4) | 0.18 | 0.27 | 1.20 | 0.71 | 2.02 | 0.5 |
| No. unplaced last season (cont 0-11) | -0.05 | 0.07 | 0.95 | 0.83 | 1.08 | 0.4 |
| No 1^st^ place in lifetime (cont 0-6) | 0.25 | 0.10 | 1.28 | 1.05 | 1.57 | 0.006 |
| No 2nd place in lifetime (cont 0-9) | 0.28 | 0.08 | 1.32 | 1.14 | 1.53 | <0.001 |
| No 3^rd^ place in lifetime (cont 0-14) | 0.15 | 0.07 | 1.17 | 1.02 | 1.34 | 0.03 |
| No 4^th^ place in lifetime (cont 0-11) | 0.24 | 0.07 | 1.27 | 1.10 | 1.47 | 0.001 |
| No unplaced lifetime (cont 0-47) | 0.07 | 0.01 | 1.08 | 1.05 | 1.11 | <0.001 |
| Average horse weight (mean 1103, S.D. 68.6) (per 10 kg) | -0.03 | 0.02 | 0.97 | 0.93 | 1.01 | 0.2 |
| Peak horse weight (per 10 kg) | -0.01 | 0.02 | 0.99 | 0.95 | 1.03 | 0.6 |

Cont = modelled as a continuous variable, range shown in brackets. Ref = reference category. HK = Hong Kong
