# Supplementary material for: Horse–, training– and race–level risk factors for palmar/plantar osteochondral disease in the racing Thoroughbred
Source: Equine Vet J. 2013 Feb 20;45(5):582–6. doi: 10.1111/evj.12038 (PMC3883097; doi:10.1111/evj.12038)
Supplement: Table S2 — Training variables examined and their relationship with grade of palmar/plantar osteochondral disease (POD) from univariable multilevel, multinomial, ordered proportional odds models in 158 Thoroughbred racehorses in Hong Kong. [file evj0045-0582-sd2.doc]

**Supplementary Item 2:**

Training variables examined and their relationship with grade of POD from univariable multilevel, multinomial, ordered proportional odds models in 158 (1240 condyles) Thoroughbred racehorse in Hong Kong. Outcome is categories of POD grade 0 to 3. POD grade 0 was treated as the reference category therefore negative coefficients (odds ratio <1) indicate that the probability of being in the lower categories is increased and positive coefficients (odds ratios >1) indicate that the probability of being in the higher categories is increased. For brevity the separate intercepts for each grade of POD and the within horse variance estimate for each analysis are not shown, however horse was included as a random effect in all analyses.

| **Variable** | **Coefficient** | **Standard Error** | **Odds ratio** | **Lower 95% CI** | **Upper 95 CI** | **P -value** |
| --- | --- | --- | --- | --- | --- | --- |
| Total career days (from first track work to retirement) (cont) | 0.13 | 0.03 | 1.13 | 1.07 | 1.20 | <0.001 |
| Total number barrier trials over lifetime (cont) | 0.10 | 0.04 | 1.11 | 1.03 | 1.19 | 0.009 |
| Total distance barrier trials (in 1000 metres) (cont) | 0.10 | 0.03 | 1.10 | 1.03 | 1.18 | 0.003 |
| Total number gallops over lifetime (cont) | 0.01 | 0.002 | 1.01 | 1.01 | 1.01 | <0.001 |
| Total distance gallops (in 1000 metres) (cont) | 0.01 | 0.002 | 1.01 | 1.01 | 1.02 | <0.001 |
| Swimming in training |  |  |  |  |  |  |
| No | Ref |  | Ref |  |  |  |
| Yes | 2.10 | 1.00 | 8.17 | 1.14 | 58.43 | 0.04 |
| Age at first track work (cont) | 4.10 | 0.30 | 60.34 | 33.78 | 107.79 | 0.2 |
| Age at first track work (cat) |  |  |  |  |  |  |
| ≤2 years | Ref |  | Ref |  |  |  |
| > 2 - ≤3 years | 0.39 | 0.35 | 1.47 | 0.74 | 2.93 | 0.3 |
| ≥ 4 years | 0.98 | 0.79 | 2.66 | 0.57 | 12.48 | 0.2 |
| Total number seasons galloping (cont) | 0.43 | 0.10 | 1.53 | 1.26 | 1.87 | <0.001 |
| Average gallops per season in lifetime (cont) | 0.08 | 0.02 | 1.08 | 1.05 | 1.11 | <0.001 |
| Total number gallops in last season before euthanasia or retirement (1-73) (cont) | 0.03 | 0.01 | 1.03 | 1.01 | 1.05 | 0.006 |
| Total number barrier trials in last season before euthanasia or retirement . | -0.18 | 0.12 | 0.83 | 0.66 | 1.04 | 0.1 |
| Between-gallop intervals current season <1 week | 0.04 | 0.01 | 1.04 | 1.01 | 1.06 | 0.007 |
| Between-gallop intervals current season 1-2 weeks | 0.10 | 0.05 | 1.11 | 1.01 | 1.22 | 0.04 |
| Between-gallop intervals current season 2-4 weeks | 0.19 | 0.11 | 1.21 | 0.97 | 1.50 | 0.09 |
| Between-gallop intervals current season 4-6 weeks | -0.30 | 0.29 | 0.74 | 0.42 | 1.31 | 0.3 |
| Between gallop intervals current season >7 weeks | 0.07 | 0.30 | 1.07 | 0.60 | 1.93 | 0.8 |
| Between-gallop intervals <1 weeks average per season over lifetime | 0.08 | 0.02 | 1.08 | 1.04 | 1.13 | <0.001 |
| Between-gallop intervals 1-2 weeks average per season over lifetime | 0.30 | 0.07 | 1.35 | 1.17 | 1.57 | <0.001 |
| Between-gallop intervals 2-4 weeks average per season over lifetime | 0.29 | 0.13 | 1.33 | 1.04 | 1.71 | 0.03 |
| Between-gallop intervals 4-6 weeks average per season over lifetime | -0.25 | 0.42 | 0.78 | 0.34 | 1.77 | 0.6 |
| Between-gallop intervals >7 weeks average per season over lifetime | 0.49 | 0.50 | 1.64 | 0.62 | 4.33 | 0.3 |

Cont = modelled as a continuous variable, range shown in brackets. Ref = reference category. HK = Hong Kong
